# Identification of a Nonstructural DNA-Binding Protein (DBP) as an Antigen with Diagnostic Potential for Human Adenovirus

**DOI:** 10.1371/journal.pone.0056708

**Published:** 2013-03-13

**Authors:** Li Guo, Chengjun Wu, Hongli Zhou, Chao Wu, Gláucia Paranhos-Baccalà, Guy Vernet, Qi Jin, Jianwei Wang, Tao Hung

**Affiliations:** 1 MOH Key Laboratory of Systems Biology of Pathogens and Christophe Mérieux Laboratory, IPB, CAMS-Fondation Mérieux, Institute of Pathogen Biology (IPB), Chinese Academy of Medical Sciences (CAMS) & Peking Union Medical College (PUMC), Beijing, People’s Republic of China; 2 Fondation Mérieux, Lyon, France; 3 National Institute for Viral Disease Control and Prevention, Chinese Center for Disease control and Prevention, Beijing, People’s Republic of China; The University of Hong Kong, Hong Kong

## Abstract

**Background:**

Human adenoviruses (HAdVs) have been implicated as important agents in a wide range of human illnesses. To date, 58 distinct HAdV serotypes have been identified and can be grouped into six species. For the immunological diagnosis of adenoviruses, the hexon protein, a structural protein, has been used. The potential of other HAdV proteins has not been fully addressed.

**Methodology/Principal Findings:**

In this study, a nonstructural antigenic protein, the DNA binding protein (DBP) of human adenovirus 5 and 35 (Ad5, Ad35) - was identified using immunoproteomic technology. The expression of Ad5 and Ad35 DBP in insect cells could be detected by rhesus monkey serum antibodies and healthy adult human serum positive for Ad5 and Ad35. Recombinant DBPs elicited high titer antibodies in mice. Their conserved domain displayed immunological cross-reactions with heterologous DBP antibodies in Western blot assays. DBP-IgM ELISA showed higher sensitivity adenovirus IgM detection than the commercial Adenovirus IgM Human ELISA Kit. A Western blot method developed based on Ad5 DBP was highly consistent with (χ^2^ =  44.9, *P*<0.01) the Western blot assay for the hexon protein in the detection of IgG, but proved even more sensitive.

**Conclusions/Significance:**

The HAdV nonstructural protein DBP is an antigenic protein that could serve as an alternative common antigen for adenovirus diagnosis.

## Introduction

Viral structural proteins, which generally elicit strong host immune responses, have been widely used in vaccine and diagnostic reagent development. However, humoral and cellular immunity can also be triggered by many viral nonstructural proteins, such as by NS3, a hepatitis C virus (HCV) protein [1]; NS1, a Dengue virus protein [2]; and P27, a human immunodeficiency virus (HIV) protein [3]. Viral nonstructural proteins can stimulate unique immune responses during certain infectious processes [4]. This antigenicity and immunogenicity of viral nonstructural proteins aids vaccine design and clinical diagnosis of viral infections [4], [5].

Human adenoviruses (HAdV), members of the *Mastadenovirus* family, are non-enveloped, linear, double-stranded DNA viruses with icosahedral capsids [6]. The ∼36 kb HAdV genome encodes five early transcripts (E1A, E1B, E2, E3, and E4), three delayed early transcripts (IX, IVa2, and E2 late), and one major late transcripts, which encodes five families of mRNAs (L1 to L5) [7]. The viral structural proteins encoded by the HAdV genome include major capsid proteins (hexon, penton base, and fiber), minor capsid proteins (pIIIa, pVI, pVIII and pIX), and core proteins (pV, µ, pVII, pTP). To date, at least 58 distinct serotypes have been identified [8]. These serotypes have been grouped into six species HAdV-A to HAdV-F [8] based on their immunological, biological, and biochemical characteristics.

HAdV has been implicated as a significant agent in a wide range of human illnesses, including respiratory disease, gastroenteritis, pharyngitis, keratoconjunctivitis, meningoencephalitis, acute hemorrhagic cystitis, and hepatitis [9]. Adenoviruses most often cause acute respiratory tract infections in children [7]. These viruses typically result in subclinical infections in immunocompetent individuals, but usually do not cause permanent problems or death [7]. However, in immunocompromised patients adenovirus infections are often severe and can be fatal [7].

Diagnosis of HAdV infections is currently based on virus isolation, viral DNA detection, antigen detection, and antibody detection [7]. Hexon, the major HAdV antigen, contains genus-specific epitopes and is often used for serum antibody diagnosis [10]. Fiber knob and protein IX (pIX) are important candidate antigens for species-specific adenovirus diagnosis based on species-dependent sequences [10]. No additional HAdV antigens have been identified for viral diagnosis.

Proteomics has been widely used to analyze protein components of pathogens [11], [12]. Immunoproteomic technologies that combine 2DE-PAGE with immunoblot followed by mass spectrometry have been widely used to identify new biomarkers and antigens for potential drug targets and vaccine candidates [13]–[16]. For instance, human serum samples and meningococcal carriage strains have been used together with immunoproteomic analysis to investigate the OM (outer membrane) protein antigens [15]. These protein antigens have been associated with the development of serum bactericidal activity (SBA) to both homologous colonizing meningococci and heterologous meningococcal serogroup B strains [15].

In this study, we identified the DNA binding protein (DBP) of HAdV as antigenic to anti-HAdV antibodies in immunoproteomic analysis. The DBP, a nonstructural HAdV protein, is not incorporated into the mature virion. It possesses multiple functions during viral infection and is synthesized in large amounts in virus-infected cells [17], [18]. DBP can be cleaved into two domains using mild chymotrypsin: an N-terminal domain (for Ad5, aa 1-173) and a C-terminal domain (for Ad5, aa 174-529) [17]. Unlike the N-terminal domain, the C-terminal domain is well conserved among different serotypes and harbors the DNA binding domain [17]. The properties of DBP show its potential as an antigen for adenovirus diagnosis. We here demonstrate that DBP is an antigenic and immunogenic protein that could serve as an alternative common antigen for adenovirus diagnosis.

## Results

### Identification of HAdV DNA-binding protein as an antigen

**Figure 1:**
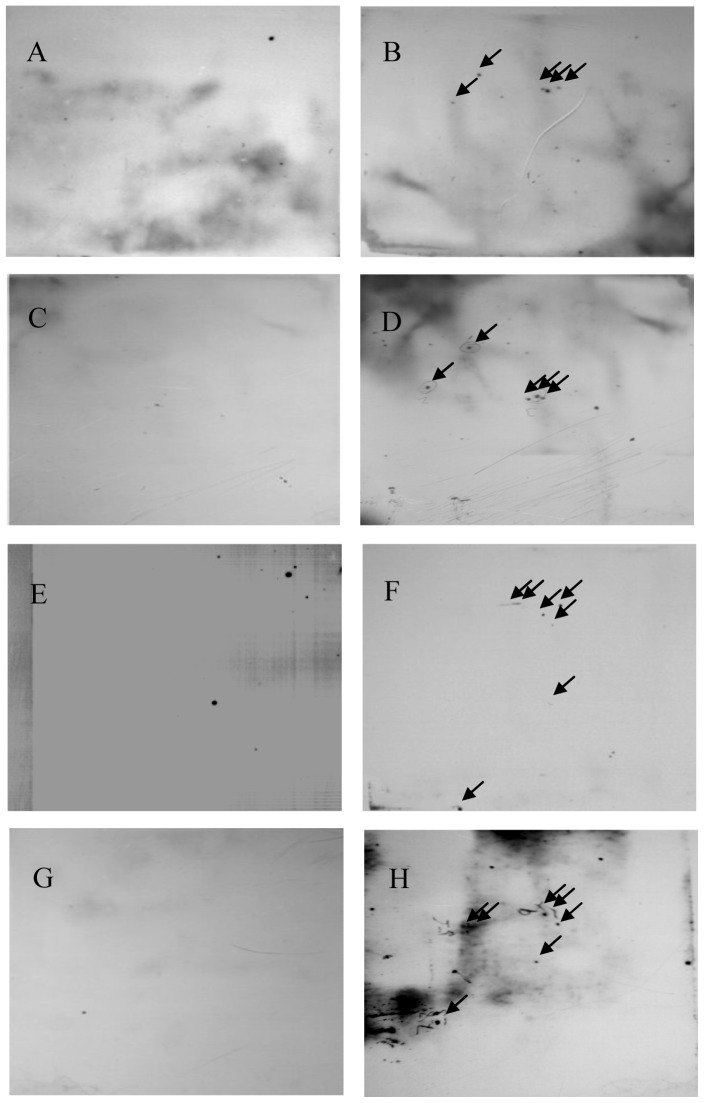
2DE SDS-PAGE/immunoblot analysis of total cellular proteins of HEK293 cells infected with Ad5 or Ad35. The total protein of Ad5 (B, D) and Ad35 (F, H) infected and mock infected (A, C, E, G) HEK293 cells were separated by 2DE SDS-PAGE. 2DE SDS-PAGE gels were transferred onto a nitrocellulose membrane. Blots were probed with in-house rhesus monkeys anti-wild Ad5 (A, B) and Ad35 antibodies (E, F) or healthy adult human serum positive for Ad5 (C, D) and Ad35 (G, H), followed by incubation with the corresponding horse radish peroxidase (HRP)-conjugated goat anti monkey or goat anti human IgG secondary antibodies.

To identify the antigenic proteins of HAdVs in humoral immunity, the total proteins of Ad5 (species C, a widely spread HAdV serotype) - and Ad35 (species B, a relatively rarely infected HAdV serotype) - infected and mock infected HEK293 cells were separated by 2DE-PAGE. These proteins were analyzed with Western blot using Ad5 and Ad35 specific antibodies produced in rhesus monkeys as well as healthy adult human serum positive for Ad5 and Ad35. For Ad5 infected cells, five proteins were identified to be differentially expressed compared to the mock infected HEK293 cells. The five proteins specifically reacted with monkeys anti-Ad5 antibodies and with healthy adult human serum positive for Ad5 (Figure 1 A-D). For Ad35 infected cells, seven differentially expressed proteins specifically reacted with anti-Ad35 antibodies and with healthy adult human serum positive to Ad35 (Figure 1 E-H).

The differentially expressed HAdV proteins identified by 2DE-PAGE and Western blot were further analyzed using MALDI-TOF/TOF. Five immunoreactive proteins of Ad5, including four structural proteins (hexon, chain F of Ad5 fiber knob, penton base, and protein VII precursor) and one nonstructural protein (DNA-binding protein, DBP) were identified (Table 1a). Similarly, seven immunoreactive proteins of Ad35 were identified, including five structural proteins (hexon, chain F of Ad35 fiber knob, penton base, protein VII precursor, and hexon associated protein IX) and two nonstructural proteins (DBP and 117aa) (Table 1b). As the nonstructural protein DBP was identified in both Ad5 and Ad35 infected HEK293 cells, our findings suggest that this nonstructural protein may function as an additional HAdV antigen.

**Table 1:** Differentially expressed proteins in Ad5 (a) and Ad35 (b) infected HEK293 cells identified by MALDI-TOF/TOF after 2DE-PAGE and Western blot analysis.

Protein name	Geninfo identifier (GI)	Molecular weight (Mw)	Isoelectric point (PI)	Sequence coverage (%)
Hexon protein	187875972	11474.7	7.33	27
Chain F of Ad5 Fiber Knob	109157255	24114.6	4.83	9
penton base	58177702	62877.6	5.53	29
protein VII precursor	58177703	21245.5	12.29	17
DNA-binding protein (DBP)	58077709	59124.5	7.75	21
Hexon protein	12053725	11474.7	7.33	25
Chain F of Ad35 Fiber Knob	189339614	24114.6	4.83	11
penton base	32967032	62877.6	5.53	26
protein VII precursor	32967033	21245.5	12.29	17
hexon associated protein IX	32967024	14185.2	9.15	13
DNA-binding protein (DBP)	32967039	58266.8	8.81	26
117 aa	32967055	13578.9	4.85	18

To evaluate DBP’s DNA sequence variation and its potential use in diagnosis, all of the available complete HAdV DBP sequences in GenBank were used for alignment analysis. Multiple-alignment analyses showed that the N-terminal domain (aa 1-173) identity was 9.2-21.4%, whereas the C-terminal domain (aa 174-529) identity was 55.9-68.5% among the strains of the six HAdV species. Alignment results also showed that amino acid sequences have higher identities within the same species (Table S1).

### Verification on the antigenicity of DBP

**Figure 2:**
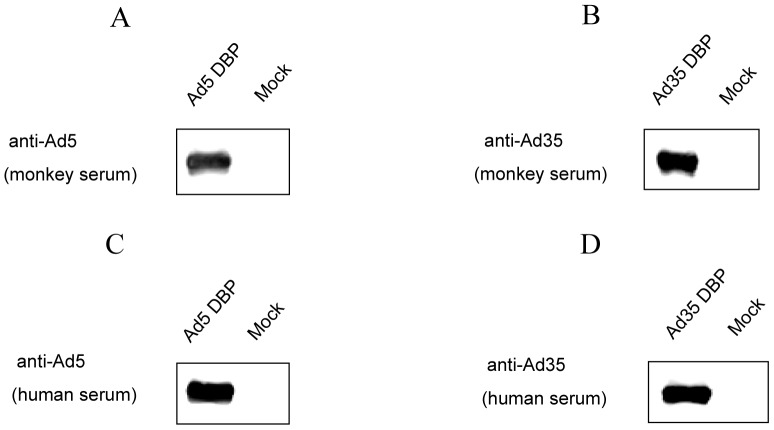
Antigenicity of Ad5 and Ad35 DBP. The Ad5 and Ad35 DBP were expressed in insect cells. Western blot analysis were performed to determine the antigenicity of Ad5 and Ad35 DBP using corresponding anti-DBP serum (1:1,000 dilutions) derived from rhesus monkeys (A and B) and anti-Ad5 and Ad35 serum (1∶400 dilutions) derived from healthy adult volunteers (C and D).

To assess the antigenicity of the HAdV nonstructural protein DBP, Ad5 DBP and Ad35 DBP were expressed in insect cells using the Bac-to-Bac® Baculovirus Expression System. rDBP proteins were purified using a HisTrap HP column and then analyzed by Western blot using rhesus monkeys serum against Ad5 or Ad35 (Figure 2 A, B). When human serum from healthy adult volunteers that were positive for Ad5 and Ad35 were used in Western blot analysis, a band specific for either Ad5 or Ad35 DBP was detected (Figure 2 C, D). These data suggest that Ad5 and Ad35 DBPs are antigenic proteins.

### Antigenic characterization of the purified rDBP proteins

**Figure 3:**
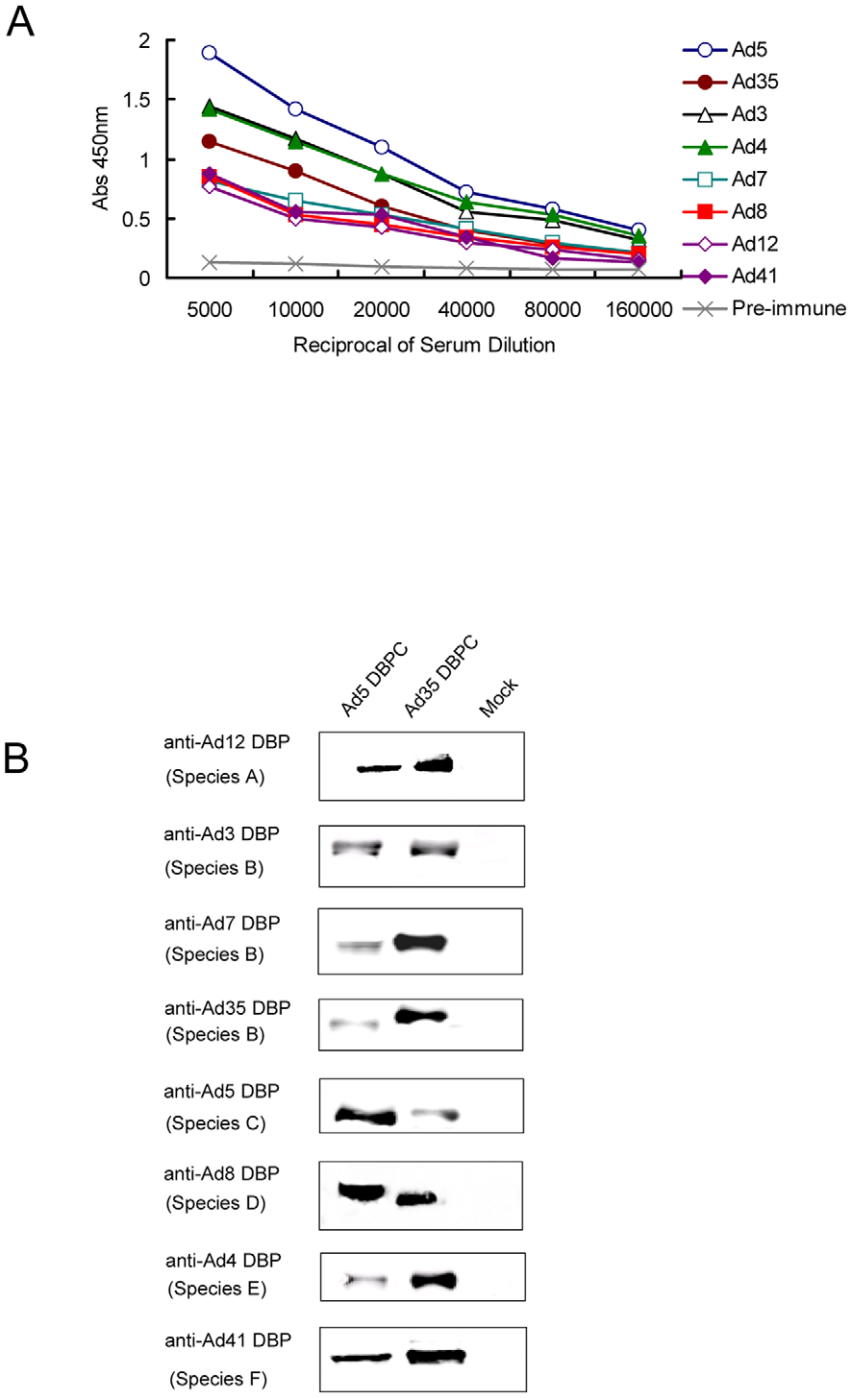
Antigenic characterization of DBPs of different HAdV species. (A) Titers of IgG antibody against different HadV species [species A (Ad12), species B (Ad3, Ad7, and Ad35), species C (Ad5), species D (Ad8), species E (Ad4), and species F (Ad41)] in mice sera were analyzed. The titers of mice sera were determined as a series of two-fold dilutions by ELISA. (B) The immunological cross-reactivity was analyzed between Ad5 DBPC/Ad35 DBPC and anti-Ad12 DBP, anti-Ad3 DBP, anti-Ad7 DBP, anti-Ad35 DBP, and anti-Ad5 DBP, anti-Ad8 DBP, anti-Ad4 DBP, and anti-Ad41 DBP mice serum by Western blot.

To test the immunogenicity of DBP, mice were immunized three times with purified rDBP of at least one representative of each species, including species A (Ad12), species B ( Ad3, Ad7, and Ad35), species C (Ad5), species D (Ad8), species E (Ad4), and species F (Ad41). After three rounds of immunizations, specific IgG antibodies against these DBPs were elicited effectively. The titers reached 1∶80,000∼1∶160,000 (Figure 3A). These results indicate that DBP of each HAdV species are strongly immunogenic.

To evaluate the cross-reactions of DBPs belonging to different species, the purified conserved domains of DBP of Ad5 (Ad5 DBPC) and Ad35 (AD35 DBPC) were subjected to Western blot analysis using in-house murine antibodies against DBPs of different HAdV species (Ad12, Ad3, Ad7, Ad35, Ad5, Ad8, Ad4, or Ad41). Results showed that Ad5 DBPC and Ad35 DBPC can be specifically recognized by homologous and heterogonous mouse polyclonal antibodies (Figure 3B), suggesting that immunological cross-reactions exist among DBP from different HAdV species.

### Performance of DBP-IgM ELISA and DBP-IgG Western blot for HAdV antibody detection

The strong antigenicity of Ad5 DBP prompted us to develop an ELISA and Western blot assay, using Ad5 DBPC as an antigen in order to evaluate its performance as a potential diagnostic compound for HAdV IgM and IgG antibodies.

First, we performed IgM ELISA to detect IgM antibodies against DBPC (DBP-IgM ELISA). Acute-phase serum samples were obtained from 50 children with HAdV infection from day 1 to day 5 after onset of fever. All children were positive for HAdV based on PCR analysis. A commercial Adenovirus IgM Human ELISA Kit (Abcam, Cambridge, UK) was used as a reference. DBPC IgM antibodies were detected in 35 samples out of the 50 tested samples using the DBP-IgM ELISA assay (Table 2). The positive rate of DBP IgM was significantly higher (1.5-fold) than that of the structural protein IgM detected with the Adenovirus IgM Human ELISA Kit (χ^2^ = 4.97, *P*<0.05), indicating a higher sensitivity of the DBP-IgM ELISA assay.

**Table 2:** Comparison of DBP-IgM ELISA and commercial Adenovirus IgM Human ELISA Kit in the diagnosis of HAdVs infection related to number of days after onset of fever.

Days after onset of fever	No. of samples	DBP-IgM ELISA	Adenovirus IgM Human ELISA Kit
1	13	11^a^ (84.6)^b^	7 (53.8)
2	5	4 (80)	3 (60)
3	11	6 (54.5)	4 (36.4)
4	8	4 (50)	4 (50)
5	13	10 (76.9)	5 (38.5)
Total	50	35 (70)	23 (46)

a. Number of positive samples.

b. Percentage of positive samples.

Next, we performed a Western blot analysis for Ad5-IgG DBP using the hexon protein as a reference. A total of 104 healthy human sera were screened for Ad5 IgG antibody prevalence. We found that 101 of the 104 sera (97.1%) were positive for both hexon and DBPC antibodies, demonstrating a good agreement (98.1%) between the two targets (Table 3). Statistical analysis showed that the results of the two methods were significantly related (χ^2^ = 44.9, *P*<0.01), and did not display differences (χ^2^ = 0.5, *P*>0.05). The sensitivity and specificity of the DBP-IgG Western blot versus those of the hexon Western blot were 99% and 66.7%, respectively. Among the 100 double positive sera, 46 (46%) displayed strong reactivity with both DBPC and hexon antigens, and 17 (17%) reacted weakly with both DBPC and hexon antigen. Interestingly, 31 (31%) of the double positive sera reacted strongly with DBPC antigens but weakly with hexon antigens. In contrast, only six (6%) samples reacted strongly with hexon antigens but weakly with DBPC antigens. These results indicate that DBP can be used for IgG antibody detection against HAdV.

**Table 3:** Comparison of DBP-IgG Western blot and hexon Western blot.

	Hexon Western blot		
DBP-IgG Western blot	Positive	Negative	Total
Positive	100	1	101
Negative	1	2	3
Total	101	3	104

χ^2^  =  44.9, *P*<0.01. The sensitivity and specificity of the DBP Western blot versus the hexon Western blot was 99% and 66.7%, respectively. Calculation formula: sensitivity  =  TP/(TP+FN), specificity  =  TN/(FP+TN), where TP is the number of true positives, FN is the number of false negatives, FP is the number of false positives, and TN is the number of true negatives [36].

## Discussion

In this study, DBP, a nonstructural adenovirus protein, was shown to be an antigen by immunoproteomic approach. DBP could be used as a potential diagnostic target for human adenovirus infection given that the amino acid sequences of full length DBP are highly identical among the same species and the C-terminal domain is conserved among different species.

To demonstrate the antigenicity of DBPs, the DBPs of at least one representative serotype of each HAdV species were expressed using a baculovirus expression system and prepared high titer antibodies. Immunological cross-reactions between mouse anti-Ad5 DBP/anti-Ad35 DBP serum and heterologous adenovirus DBP were observed. The conservation of the DBP C-terminal domain (DBPC) may account for the cross-reactivity. Further development of immunoassays may be necessary to detect antibodies or antigens of different species of human adenoviruses using Ad5 or Ad35 DBP.

DBP plays important roles in many steps in the adenovirus life cycle, including in DNA replication, transcription, mRNA stability, host range specificity, recombination, and in virus assembly [19]–[22]. DBP has a high abundance in adenovirus infected cells - about 2×10^7^ molecules of DBP per cell in the early phase of infection [18]. Furthermore, DBP has an about 100-fold higher expression level than pol and pTP [18]. Thus, it is of interest and potential use in adenovirus infection diagnosis. Indeed, some nonstructural virus proteins have been used in laboratory immunoassays for virus infection due to their unique antigenicity [23]–[27]. For examples, the influenza virus NS1 protein combined with NP and M sandwich ELISA could improve detection sensitivity [23]. Dengue virus NS1 protein has been used for early diagnosis and the sensitivity of the NS1 IgM ELISA was higher than that of RT-PCR [24]. In the immunoasssays of anti-HCV IgG detection, the NS4 region was used as the first-generation assays, which could identified anti-HCV IgG in approximately 80% of patients with posttransfusion hepatitis [28]. Second- and third-generation assays used a multiantigen format including NS4, NS3, and core regions, which markedly improved the sensitivity and specificity of the diagnosis [25]–[27].

Our results showed that DBP-IgM ELISA is more sensitive than a commercial Adenovirus IgM Human ELISA Kit, which mainly uses the structural proteins of adenoviruses as an antigen. Our DBPC-IgG Western blot was consistent with the hexon-based Western blot but had higher sensitivity. Taken together, these finding suggest that DBP could function as an alternative antigen for the detection of HAdV antibodies and could serve as a novel antigen for early phase diagnosis of adenovirus infection.

In this study, the number of negative samples was low because of the low overall number of the samples detected and the high prevalence of HAdVs IgG in the population. To evaluate the specificity and selectivity of the antigen further with respect to other HAdV species, large scale samples in more detailed studies are needed. Moreover, it will be necessary to assess the immunological cross-reactivity of anti-DBP IgM antibody in ELISA assays further to confirm the sequence alignment and Western blot results using anti-DBP IgG antibody for the immunological cross-reactivity of anti-DBP IgM antibody in the ELISA.

Adenoviruses vectors have attracted great interest in gene therapy. However, a major limitation is the host immune response against adenoviruses, which prevents sustained expression of the foreign genes and has raised some concerns about the safety of HAdV vectors [29], [30]. The neutralizing antibodies of HAdV are primarily directed against the hexon protein [31]. Gaining insights into the immunogenicity of DBP and prevalence of DBP antibodies in humans will be helpful for the design and improvement of adenoviral vectors to investigate the effects of anti-DBP immune responses on the safety and efficacy of adenoviral vectors in future vaccine studies.

In summary, using immunoproteomic approaches, the nonstructural protein of Ad5 and Ad35, DBP, has been identified in this study as being immunogenic. DBP-IgM ELISA and DBP-IgG Western blot analyses suggest that DBP can serve as a diagnostic tool for HAdV surveillance. Our findings also provide a better understanding of the immunological mechanisms of human adenoviruses.

## Materials and Methods

### Cell culture of wild type Ad5 and Ad35

Human embryonic kidney (HEK293) cells were infected with wild type Ad5 and Ad35 at a multiplicity of infection (MOI) of 5. After one hour adsorption, the inocula were removed and maintained in Dulbecco’s modified Eagle medium (DMEM) containing 2% fetal bovine serum (FBS;Invitrogen, Carlsbad, CA).

### Two-dimensional immunoblot analysis of Ad5 and Ad35 proteins

Wild type Ad5 and Ad35 infected HEK293 cells were collected 36 hours post infection (hpi) and lysed in RIPA buffer (Roche, Indianapolis, IN). Roughly 75 µg total protein was first separated by isoelectric focusing (IEF) over a pH range of 3-10 using precast first-dimension drystrip (GE Healthcare, Waukesha, WI). The first-dimension strips were equilibrated in equilibration buffer [50 mM Tris (pH 8.8), 6 M urea, 20% glycerol, 2% SDS] plus 1% DTT for 15 minutes, and then equilibrated in equilibration buffer plus 4% iodoacetamide for 15 minutes. The equilibrated first-dimension strip was loaded on a 12% SDS-PAGE. One gel was fixed and stained using 0.1% Coomassie Blue Brilliant R-250 for mass spectrometry analysis, and the other duplicate gel was transferred onto nitrocellulose membrane (Pall, Port Washington, NY) for subsequent immunoblot analysis.

### Western blot

Infected cells were collected and lysed in RIPA buffer. Aliquots of cell lysates (about 12 µg total cell proteins) were loaded onto a 12% SDS-PAGE gel or 2DE-PAGE to separate the proteins. The gels were transferred to a nitrocellulose membrane (Pall) and blocked with 5% nonfat dry milk. In-house anti-wild Ad5 and Ad35 rhesus monkey antibodies or healthy adult human sera positive for Ad5 and Ad35 were applied, followed by incubation with the corresponding horse radish peroxidase (HRP)-conjugated goat anti monkey or goat anti human IgG secondary antibodies (Sigma, St. Louis, MO). The signal was detected by an ECL detection system (Thermo Scientific, Rockford, IL).

### MALDI-TOF/TOF analyses

Protein spots of interest based on the 2DE-PAGE and immunoblot results were excised from the Coomassie Blue-stained gels. MALDI-TOF/TOF (ABI4800 plus; Applied Biosystems, Foster, CA) analyses were performed at the Beijing Proteome Research Center (Beijing, China). The proteins were unambiguously identified by searching against the NCBInr virus database using MASCOT 2.0 program (Matrix Science, Boston, MA).

### Expression of DBP protein and animal immunization

The full-length E2A gene of Ad5 was amplified from the pAdEasy-1 (Stratagene, Cedar Creek, TX) and that of Ad35 from the pAd35-backbone vector [32]. Ad5 E2A is 1,590 bp long [nucleotide (nt) 22443-24032 according to Ad5 genome sequence, GenBank accession number AC-000008], and Ad35 E2A is 1,557 bp long (nt21860-23416 according to Ad35 strain 35p, GenBank accession number AY271307). The PCR products were cloned into the baculovirus transfer vector, pFastBacHT A (Invitrogen). The conserved domain of Ad5 E2A (Ad5 E2AC, nt 520-1590 based on the Ad5 E2A gene) and Ad35 E2A (Ad35 E2AC, nt 478-1557 based on Ad35 E2A gene) were cloned into baculovirus vector pFastBacHT A (Invitrogen).

Additionally, the Ad3 E2A gene (1,554 bp in length, nt 22,006-23,559 according to Ad3 genome sequence, GenBank accession number AY599834), Ad4 E2A gene (1,539 bp in length, nt 21,774-23,312 according to Ad4 genome sequence, GenBank accession number AY487974), Ad7 E2A gene (1,554 bp in length, nt 22,225-23,778 according to Ad7 genome sequence, GenBank accession number AY495969), Ad8 E2A gene (1473bp in length, nt 21280-22752 according to Ad8 genome sequence, GenBank accession number AB746853), Ad12 E2A gene (1455 bp in length, nt 21215-22669 according to Ad12 genome sequence, GenBank accession number X73487), and the Ad41 E2A gene (1425 bp in length, nt 21054-22478 according to Ad41 genome sequence, GenBank accession number HM565136) were synthesized by Sangon Biotech (Shanghai, China) and cloned into the baculovirus vector pFastBacHT A (Invitrogen). The recombinant baculoviruses were generated in Sf9 cells using the Bac-to-Bac® Baculovirus Expression System (Invitrogen) protocol provided by the manufacturer. High Five cells (Invitrogen) were infected with recombinant baculoviruses carrying Ad12 DBP, Ad3 DBP, Ad7 DBP, Ad35DBP, Ad5 DBP, Ad8 DBP, Ad4 DBP, or Ad41 DBP at an MOI of 5. The infected cells were collected three days post infection and purified using a HisTrap HP 5 ml column (GE Healthcare, Waukesha, WI). Purified recombinant DBP (rDBP) and DBPC (rDBPC) were confirmed by Western blot analysis using an anti-6×Histidine monoclonal antibody (Sigma). The concentrations of all purified proteins were determined using the BCA Protein Assay Reagent Kit (Thermo Scientific). Proteins were stored at -70°C prior to use.

### Animal immunization

To evaluate the immunogenicity of DBP, 6- to 8-week old female BALB/c mice were intraperitoneally injected with 100 µg purified full length rDBP proteins of Ad12, Ad3, Ad7, Ad35, Ad5, Ad8, Ad4, or Ad41 in Freund’s complete adjuvant (Sigma). The mice were boosted twice at 2-week intervals with 50 µg purified rDBP proteins in Freund’s incomplete adjuvant (Sigma). Serum samples were collected prior to each immunization and 2 weeks following the last immunization. Specific serum immunoglobulin G (IgG) antibodies were determined by ELISA. This study was carried out in strict accordance with Chinese government's animal experiment regulations. All the animal experiments were performed in the facilities of the Institute of Laboratory Animal Sciences (ILAS), Chinese Academy of Medical Sciences (CAMS). All the experimental procedures were approved (permit number SYXKJ2009-0017) and supervised by the Animal Protection and Usage Committee of ILAS, CAMS.

### ELISA

The anti-rDBP IgG antibodies produced in immunized mice were measured by ELISA assay, as described elsewhere [33]. Purified rDBPs were used as coating antigens (0.25 µg/mL) for the ELISA assay.

For DBP-IgM ELISA, 96-well microtiter plates (Costar, Acton, MA) were coated with 25 ng purified Ad5 rDBPC per well at 4°C overnight. The plates were blocked with 1% (w/v) bovine serum albumin (BSA, Sigma) in PBS at 37°C for 2h; afterwards they were washed three times with 0.05% Tween 20 in PBS (PBS-T). Human serum samples diluted 1∶100 were then added to the wells and incubated for 1h at 37°C. After washing five times with PBS-T, HRP-conjugated goat anti-human IgM (Sigma) was added to the plates at a dilution of 1∶40,000 and the plates were then incubated at 37°C for 1h. Plates were washed five times and developed with 100 µl/well substrate solutions A and B (Wantai Biotech Corp. Beijing, China). The reaction was stopped by the addition of 50 µl of 2 M sulfuric acid to each well. The absorbance at 450 nm was determined using the multifunctional microplate reader SpectraMax M5 (Molecular Devices, Sunnyvale, CA).

To determine the cut-off values for the DBP-IgM ELISA, negative sera samples collected from infants aged 0-6 months who visited Beijing Children’s Hospital for regular health check-ups were identified using Western blot analysis against the hexon protein and PCR analysis targeting the hexon gene. As the cut-off values we used the mean absorbance at 450 nm of the negative sera plus three-folds the standard deviation.

### Serum samples

Serum specimens were obtained from 104 healthy individuals to evaluate the DBP-IgG Western blot developed in this study. All these samples were collected from the Beijing Blood Center during routine physical examinations. In addition, acute-phase serum samples were collected from 50 children (median age 14 months; range of 1 month to 13 years) with acute low respiratory tract infections (ALRTIs) when they were hospitalized at the Beijing Children’s Hospital. The DNA of HAdV was detected in the nasopharyngeal aspirates of these 50 patients at the time of admission by PCR analysis using primers targeting the hexon gene to amplify a 301 base pair (bp) fragment, as described elsewhere [34]. All serum samples were stored at -80°C prior to use.

Written informed consent was obtained from all participants or guardians on behalf of children. This study was approved by the ethical review committee of the Institute of Pathogen Biology, Chinese Academy of Medical Sciences, and by Beijing Children’s Hospital.

### Phylogenetic analysis

Multiple sequence alignments were analyzed using MEGA 4.0 software [35] based on full length amino acid sequences of all the available HAdV serotype DBP sequences.

### Statistical Analysis

The comparison of DBP-IgM ELISA with a commercial Adenovirus IgM Human ELISA Kit and the DBP-IgG Western blot analysis with the hexon Western blot analysis were evaluated using the χ^2^-test using SPSS software.

## Supporting Information

Table S1 **Pairwise nucleotide and amino acid sequence comparisons of DBP within the same HAdVs species.**
(DOC)
