# Supplementary material for: Identification of a Nonstructural DNA-Binding Protein (DBP) as an Antigen with Diagnostic Potential for Human Adenovirus
Source: PLoS One. 2013 Mar 13;8(3):e56708. doi: 10.1371/journal.pone.0056708 (PMC3596362; doi:10.1371/journal.pone.0056708)
Supplement: Table S1 — Pairwise nucleotide and amino acid sequence comparisons of DBP within the same HAdVs species. (DOC) [file pone.0056708.s001.doc]

TABLE S1. Pairwise nucleotide and amino acid sequence comparisons of DBP within the same HAdVs species

|  | Species A | Species B1 | Species B2 | Species C | Species D | Species E | Species F |
| --- | --- | --- | --- | --- | --- | --- | --- |
| Nucleotide sequence identity (%) | 80.2–100 | 95.4 –100 | 98.2 –100 | 96.7–98.7 | 93.1–100 | 96.3–100 | 65.7–71 |
| Amino acid sequence identity (%) | 80.8–100 | 96.3–100 | 99 –100 | 97.7–100 | 95.3–100 | 97.8–100 | 62.8–72.1 |
